# Fumaric Acid and Slightly Acidic Electrolyzed Water Inactivate Gram Positive and Gram Negative Foodborne Pathogens

**DOI:** 10.3390/microorganisms3010034

**Published:** 2015-02-12

**Authors:** Charles Nkufi Tango, Ahmad Rois Mansur, Deog-Hwan Oh

**Affiliations:** Department of Food Science and Biotechnology, College of Biotechnology and Bioscience, Kangwon National University, Hyoja 2 dong, Chuncheon 200-701, Korea; E-Mails: charlynkufi2@yahoo.fr (C.N.T.); rais.ahmad07@yahoo.com (A.R.M.)

**Keywords:** slightly acidic electrolyzed water, Fumaric acid, inactivation, pure culture, foodborne pathogens

## Abstract

Sanitizing effectiveness of slightly acidic electrolyzed water (SAEW) and fumaric acid (FA) at different dipping temperatures (25–60 °C), times (1–5 min), and concentrations (5–30 ppm for SAEW and 0.125%–0.5% for FA) on pure cultures of two Gram positive pathogens *Staphylococcus aureus* (SA) and *Listeria monocytogenes* (LM) and two Gram negative pathogens *Escherichia coli* O157:H7 (EC) and *Salmonella* Typhimurium (ST) was evaluated. FA (0.25%) showed the strongest sanitizing effect, demonstrating complete inactivation of EC, ST*,* and LM, while SA was reduced by 3.95–5.76 log CFU/mL at 25–60 °C, respectively, after 1 min of treatment. For SAEW, the complete inactivation was obtained when available chlorine concentration was increased to 20 ppm at 40 °C for 3 and 5 min. Moreover*,* Gram positive pathogens have been shown to resist to all treatment trends more than Gram negative pathogens throughout this experiment. Regardless of the different dipping temperatures, concentrations, and times, FA treatment was more effective than treatment with SAEW for reduction of foodborne pathogens. This study demonstrated that application of FA in food systems may be useful as a method for inactivation of foodborne pathogens.

## 1. Introduction

Bacterial foodborne illnesses occur when food contaminated with foodborne pathogens is consumed and the bacteria continue to grow in the intestines, with illness lasting throughout the production of toxin. More than 90% of the cases of foodborne illness are caused by *Staphylococcus aureus* (SA), *Salmonella* spp., *Listeria monocytogenes* (LM), and entero-hemorrhagic *Escherichia coli* O157:H7 (EC). These bacteria are commonly found on many raw foods and have been recognized as foodborne pathogens which can pose a high health risk, often with lethal consequences [1]. These foodborne pathogens can be controlled following three principal approaches developed by Suzuki *et al.* [2]: prevention of microbial contamination (handling practices), halting of microbial growth, and protection against pathogens using disinfection. Under these approaches, there is a demand for the optimization of inactivation treatments in order to ensure the microbiological safety of different foods, agricultural products and food-handling equipment. Treatments should not only induce significant inactivation of the inherent pathogenic and spoilage microorganisms, but also maintain the native sensorial properties of food while leaving no residues, be acceptable to the consumer and legislator, and be environmentally friendly [3].

Electrolyzed water (EW) has been reported to have strong bactericidal activity against most pathogenic bacteria and is recognized as a safe, quick method which can be produced on-site, while its economic cost makes it a feasible antimicrobial treatment [4,5]. It is generated by passing a current of electricity through a dilute saltwater solution using a membrane cell to produce strong acidic electrolyzed water or a dilute hypochloric acid using a non-membrane cell to produce slightly acidic electrolyzed water (SAEW). With a range of available chlorine, 10–30 mg/L and pH 5.0–6.5 SAEW have been authorized for use as food sanitizer by the Japanese Ministry of Health and Welfare [1]. SAEW has low amounts of available chlorine and the pH values range from 5.0 to 6.5, which reduces the impact of corrosion of the processing equipment and irritation of skin. Thus, it is considered as an environmentally friendly sanitizer compared to other chemical disinfectants. The effectiveness of SAEW as a sanitizer has been demonstrated for the inactivation of EC, LM, *Salmonella* spp. and SA on different products, including fruits and vegetables [6], on beef and pork meats [4], eggs and poultry [7], and seafood [8]. It has also proved to be effective for the disinfection of the surfaces of different food processing equipment [9].

Many organic acids such as acetic acid (AA), citric acid (CA), and lactic acid (LA) have been extensively evaluated, showing strong sanitizing effects on SA, EC, LM, and *Salmonella* spp. [7,10,11]. However, the inhibitory effect of organic acids depends on the undissociated form, as well as its ability to donate hydrogen ions in an aqueous system [12]. Fumaric acid (FA) is stronger than citric acid in terms of ionization constant and pH at constant concentrations, and is one of the most acidic of the solid food acids [13]. FA is since designated as generally recognized as safe by the U.S. Food and Drug Administration and for human consumption it should be used at a level not to exceed the amount required to accomplish their intended effects [1]. Treatment with 50 mM of FA for 10 min caused a 2 log reduction in the populations of EC and *Salmonella* Typhimurium (ST) attached to fresh-cut lettuce [14], and FA (10%) had a significant bactericidal effect when added to apple cider [15]. Podolak *et al.* [13] studied the successful reduction of LM, EC and ST on beef sanitized with FA during storage.

The effectiveness of a sanitizer at reducing the bacterial populations varies with the sensitivity of the target organism, concentration, contact time, and specially dipping temperature [16]. With some food products such as fresh produce, usually the inactivation is carried out at room temperature. However, other products such as meat carcasses, the washing needs high temperatures to enhance the antimicrobial effect of sanitizer. Additionally, the pathogens can grow, persist and form biofilm on food processing plants. The sanitizers used to wash these plants may need high temperatures to improve the sanitizer’s antimicrobial effect. Therefore, the current work was undertaken to evaluate the effectiveness of SAEW and FA for the inactivation of two Gram positive microorganisms (SA and LM) and two Gram negative microorganisms (EC and ST) using a wide range of temperature (25–60 °C) and to compare their sanitizing effects under different physicochemical conditions.

## 2. Materials and Methods

### 2.1. Bacterial Strain and Preparation of Inoculums

The strains of EC (ATCC 43894), SA (ATCC 12598), LM (ATCC 19115), and ST (ATCC 14028) were obtained from the University of Georgia (Griffin, GA, USA) and maintained at −70 °C in tryptic soy broth (TSB, Difco, Sparks, MD, USA) with 10% glycerol. Stock cultures of each strain were transferred into tryptic soy broth (TSB, Difco) and incubated for 24 h at 35 °C. Following incubation, 10 mL of each culture was sedimented by centrifugation (3000× *g* for 10 min at 4 °C), washed twice with 0.1% buffered peptone water (BPW, Difco) and resuspended in 10 mL of the same solution to obtain final cell concentrations of 10^8−9^ CFU/mL. The bacterial population in each culture was confirmed by plating 0.1 mL aliquots of the appropriately diluted culture on tryptic soy agar (TSA, Difco) and incubating the plates at 35 °C for 24 h.

### 2.2. Preparation of Slightly Acidic Electrolyzed Water and Fumaric Acid

The SAEW used in this study was provided by Cosmic Round Korea Co., Gyeonggi-do, Korea. The SAEW had a pH of 6.29, an oxidation reduction potential (ORP) of 820–934 mV and available chlorine concentration (ACC) of 30 ppm. The SAEW was then diluted in distillated water to obtain SAEW samples with the ACCs of 5, 10, 20, and 30 ppm. Crystalline FA (Daejung chemicals and metals Co., LTD, Gyonggi-do, Korea) was dissolved in distillated water using a magnetic stirring and diluted to give different concentrations (0.125%, 0.25%, and 0.5%) of FA solutions (w/v). The ORP and pH values of the tested solutions were measured using a dual-scale pH meter (Accumet model 15, Fisher Scientific Co., Fair Lawn, NJ, USA) and calibrated using the commercial standard buffers at pH 4.01 and 7.00 (Mettler-Toledo, Analytical CH-8603, Schwerzenbach, Switzerland). The ACC of SAEW was determined by a colorimetric method using a digital chlorine test kit (RC-3F, Kasahara Chemical Instruments Corp., Saitama, Japan).

### 2.3. Treatment of Foodborne Pathogens with Sanitizers

To investigate the sanitizing effects of SAEW and FA for various dipping temperatures, concentrations and times, the experiments were performed by adding 1 mL of each bacterial suspension into sterile screw-cap tubes containing 9 mL of the sanitizer solutions, and then continuously shaking by hand to mix the resultant suspensions and enable inactivation of the bacteria [9,17]. Following treatment, 1 mL aliquots of each of the treated samples were transferred to sterile tubes containing 9 mL of neutralizing buffer solution (0.5% sodium thiosulfate plus 0.03 M phosphate buffer solution, pH 7.2 to 7.4) to stop the sanitizing activity, and the tubes were then shaken on a platform shaker at 150 rpm. After neutralization, 1 mL of the treated strain was serially diluted in 9 mL of 0.1% buffered peptone water (BPW, Difco). Following serial dilution, 1 mL of each sample was pour plated on plate count agar and incubated at 37 ± 2 °C for 24 ± 2 h. The colonies were then enumerated using heterotrophic plate count method [1] and the microbial counts expressed as log CFU/mL of sample. The difference between the initial population and the survival of pathogen population was used to estimate the Log reduction. All treatments and measurements were performed in triplicate.

To evaluate the effectiveness of SAEW and FA at varying temperatures (25, 30, 40, 50, and 60 °C) for each pathogen, the inactivation treatments were performed at an ACC of 5 ppm for SAEW and 0.25% for FA for 1 min. The best temperature was used to assess the effectiveness of SAEW (5, 10, 20, and 30 ppm) and FA (0.125%, 0.25%, and 0.5%) for 1 min. Finally, the optimum temperature and concentration conditions obtained were used to investigate the efficacy of SAEW and FA at varying contact times (1, 3, and 5 min). One water bath (VS-1205W, vision scientific Co., Ltd., Bucheon, South Korea) installed inside a laminar flow cabinet hood (1300 Series A2, thermo fisher scientific, Marietta, GA, USA) was heated to the required temperatures, and the experiments were performed under aseptic conditions.

### 2.4. Statistical Analysis

The reductions of pathogenic count (log CFU/mL) were considered for further statistical analysis to assess the differences between the effects of the tested solutions. The data (means ± standard deviation) were subjected to analysis of variance (ANOVA). Tukey’s multiple range tests were used to determine the significant difference at *p* < 0.05 using the SPSS statistical package v. 21.0 (SPSS Inc., Chicago, IL, USA).

## 3. Results

The properties (Available chlorine concentration, ORP, and pH) of the treatment solutions (SAEW and FA) used in this study are presented in Table 1. The limit of detection was 1.0 log CFU/mL, and results below the detection limit were considered as complete inactivation.

**Table 1:** pH and oxidation reduction potential values of fumaric acid at different adjusted percentages and slightly acidic electrolyzed water at different adjusted available chlorine concentrations (ACC).

Treatment Solutions	Concentration	pH	ORP (mV)
FA	0.125 ^1^	2.67 ± 095	592–596 ± 1.03
	0.25	2.42 ± 1.22	575–786 ± 1.14
	0.5	2.34 ± 2.21	568–573 ± 1.32
SAEW	5 ^2^	6.40 ± 1.28	826–859 ± 1.31
	10	5.71 ± 1.05	854–878 ± 2.05
	20	6.06 ± 1.19	848–852 ± 1.42
	30	6.29 ± 2.01	820–934 ± 1.29

^1^ FA (%); ^2^ SAEW (ppm of available chlorine concentration); ORP: oxidation reduction potential; FA: fumaric acid; SAEW: slightly acidic electrolyzed water.

### 3.1. Effect of Temperature on the Sanitizing Effect of Fumaric Acid and SAEW

**Figure 1:**
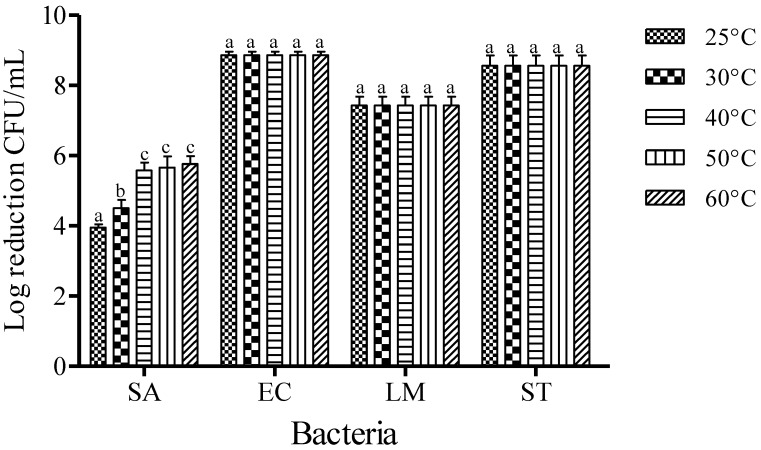
Inactivation of different foodborne pathogens treated with fumaric acid (0.25%) for 1 min at different temperatures. Vertical bars represent means of three replications ± standard deviation. Bars labeled with different letters indicate significant differences at *p* < 0.05. SA: *S. aureus*, EC: *E. coli* O157:H7, LM: *L. monocytogenes*, ST: *S.* Typhimurium. The initial level of each foodborne pathogen was 8.86, 7.68, 7.43, and 8.56 for EC, SA, LM, and ST, respectively.

The mean initial populations of EC, SA, LM, and ST used in this study were approximately 8.86 ± 0.12, 7.68 ± 0.31, 7.43 ± 0.25, and 8.56 ± 0.29 log CFU/mL, respectively. The effect of temperature on the inactivation of foodborne pathogens was examined at the concentration of 0.25% FA for 1 min of dipping (Figure 1). After treatments with FA, the reductions of SA populations were recorded to be about 3.95, 4.86, 5.58, 5.66, and 5.76 log CFU/mL at 25, 30, 40, 50, and 60 °C, respectively. However, complete inactivation was observed with the FA treatment of EC, LM, and ST at all temperatures tested after 1 min of dipping treatment. An increase of the dipping temperature from 40 to 60 °C was not observed to increase the inactivation of SA with FA treatment (*p* < 0.05).

**Figure 2:**
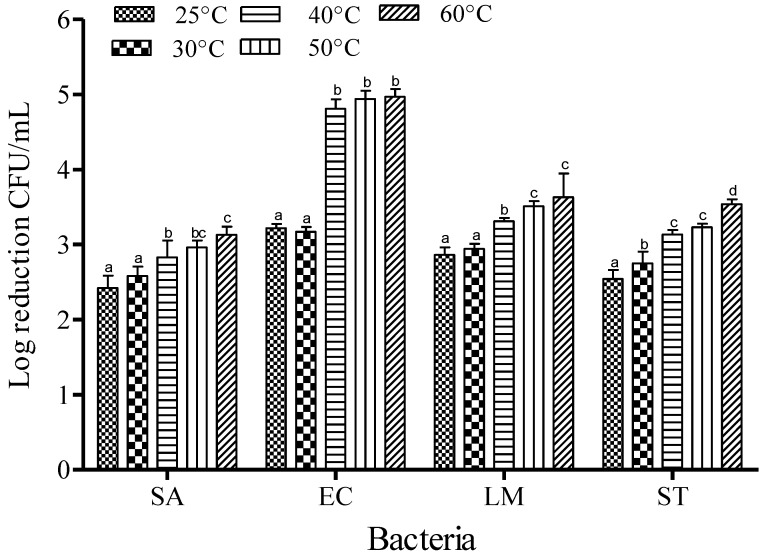
Inactivation of different foodborne pathogens treated with slightly acidic electrolyzed water (5 ppm and 1 min) at different temperatures. Vertical bars represent means of three replications ± standard deviation. Bars labeled with different letters indicate significant differences at *p* < 0.05. SA: *S. aureus*, EC: *E. coli* O157:H7, LM: *L. monocytogenes*, ST: *S.* Typhimurium. The initial level of each foodborne pathogen was 8.86, 7.68, 7.43, and 8.56 for EC, SA, LM, and ST, respectively.

For SAEW, the reduction of foodborne pathogens was studied for 1 min of dipping at an ACC of 5 ppm. The reduction of SA was recorded to be about 2.42, 2.58, 2.83, 2.96 and 3.13 log CFU/mL at 25, 30, 40, 50 and 60 °C, respectively. The trends of reduction were similar for all foodborne pathogens: populations of EC were reduced by 3.22, 3.17, 4.81, 4.94 and 4.97 log CFU/mL; LM were reduced by 2.86, 2.94, 3.31 and 3.54 log CFU/mL; and ST were reduced by 2.54, 2.75, 3.13 and 3.54 log CFU/mL at 25, 30, 40, 50 and 60 °C, respectively (Figure 2). Increasing the dipping temperature significantly enhanced the inactivation effect of SAEW (*p* < 0.05).

### 3.2. Effect of Concentration on Sanitizing Effect of Fumaric Acid and SAEW

**Figure 3:**
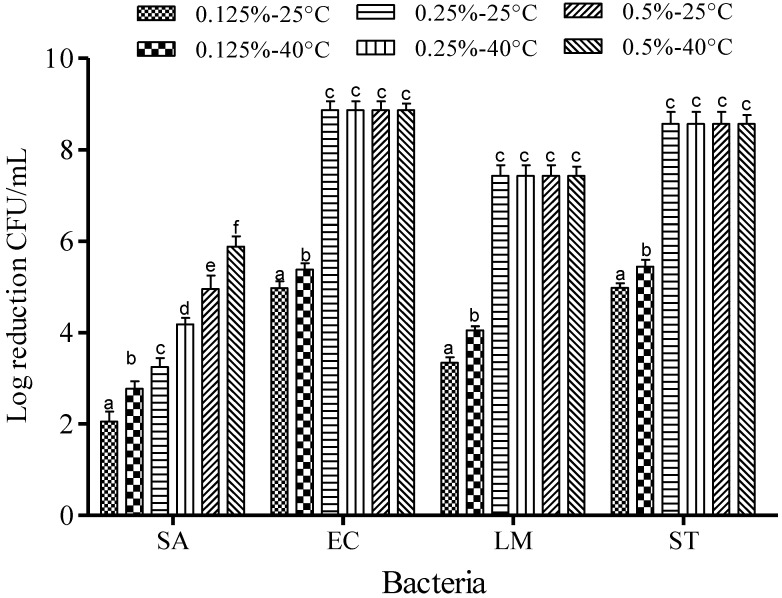
Inactivation of different foodborne pathogens treated with fumaric acid at different concentrations and temperatures for 1 min. Vertical bars represent means of three replications ± standard deviation. Bars labeled with different letters indicate significant differences at *p* < 0.05. SA: *S. aureus*, EC: *E. coli* O157:H7, LM: *L. monocytogenes*, ST: *S.* Typhimurium*.* The initial level of each foodborne pathogen was 8.86, 7.68, 7.43, and 8.56 for EC, SA, LM, and ST, respectively.

**Figure 4:**
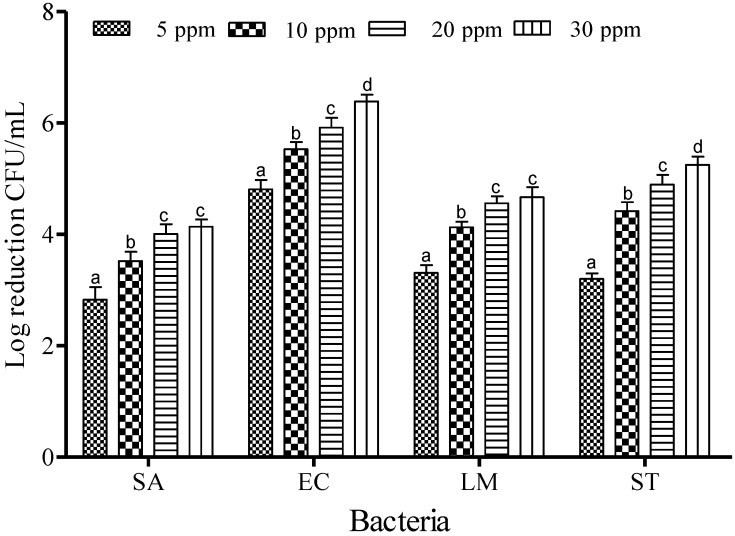
Inactivation of different foodborne pathogens treated with slightly acidic electrolyzed water (40 °C and 1 min) at different concentrations. Vertical bars represent means of three replications ± standard deviation. Bars labeled with different letters indicate significant differences at *p* < 0.05. SA: *S. aureus*, EC: *E. coli* O157:H7, LM: *L. monocytogenes*, ST: *S.* Typhimurium*.* The initial level of each foodborne pathogen was 8.86, 7.68, 7.43, and 8.56 for EC, SA, LM, and ST, respectively.

Reduction of the foodborne pathogens was observed at 25 and 40 °C while adjusting the concentration of FA (0.125%, 0.25%, and 0.5%) (Figure 3) and SAEW (5, 10, 20, and 30 ppm of ACC) (Figure 4). After treatment with FA (0.25%–0.5%), the populations of EC, LM and ST were reduced to undetectable levels after 1 min of dipping. Lower FA concentration (0.125%) reduced the respective EC, LM and ST populations by only 4.97, 3.34 and 3.20 log CFU/mL at 25 °C, and 5.38, 4.05 and 5.44 log CFU/mL at 40 °C (Figure 3). However, only about 2.05–5.88 log CFU/mL reduction of SA treated with FA (0125%–0.5%) was observed after 1 min of dipping. The effectiveness of FA increased with increasing concentration at the two different temperatures (*p* < 0.05).

Figure 4 shows the inactivation efficacy of SAEW at 40 °C. The reductions in bacterial count were about 2.83, 3.52, 4.01 and 4.14 log CFU/mL at ACCs of 5, 10, 20 and 30 ppm for SA. Similar trends of reduction were also found for all foodborne pathogens: populations of EC were reduced by 4.81, 5.53, 5.91 and 6.39 log CFU/mL; LM were 3.31, 4.13, 4.56 and 4.46 log CFU/mL; and ST were reduced by 3.2, 4.42, 4.89 and 5.25 log CFU/mL for ACC values of 5, 10, 20 and 30 ppm, respectively (Figure 4). The effectiveness of SAEW increased with increasing available chlorine at 40 °C (*p* < 0.05).

### 3.3. Effect of Dipping Time on Sanitizing Effect of SAEW

**Figure 5:**
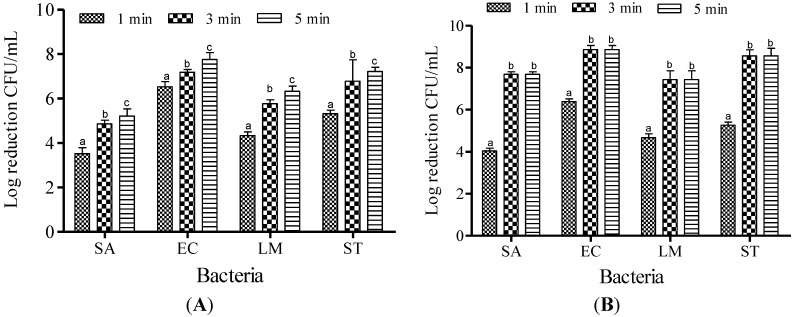
Inactivation of different foodborne pathogens treated with slightly acid low concentration electrolyzed water at 10 ppm (**A**) and 20 ppm (**B**) at 40 °C for different dipping times. Vertical bars represent means of three replications ± standard deviation. Bars labeled with different letters indicate significant differences at *p* < 0.05. SA: *S. aureus*, EC: *E. coli* O157:H7, LM: *L. monocytogenes*, ST: *S.* Typhimurium*.* The initial level of each foodborne pathogen was 8.86, 7.68, 7.43, and 8.56 for EC, SA, LM, and ST, respectively.

Increasing the ACC from 20 to 30 ppm did not result in significant (*p* < 0.05) reduction in the populations of SA and LM (Figure 4); therefore, 10 and 20 ppm were chosen to determine the effect of contact time for the potential of SAEW. The effectiveness of SAEW against SA, EC, LM and ST at 40 °C for the various dipping times (1, 3, and 5 min) are shown in Figure 5. After treatment with 10 ppm of SAEW solution, a reduction of the SA population was recorded as about 3.52, 4.86 and 5.21 log CFU/mL for 1, 3 and 5 min of dipping time, respectively. More or less similar reduction patterns were found for the populations of LM: 4.33, 5.77 and 6.33 log CFU/mL. Significant differences (*p* < 0.05) were found in SA population reductions compared to those observed for the populations of EC: 6.53, 7.18 and 7.76 log CFU/mL and ST, reduced by 5.32, 6.78 and 8.56 log CFU/mL at 1, 3 and 5 min of dipping, respectively. When the available chlorine concentration was increased to 20 ppm, complete inactivation was observed from 3 to 5 min for all examined foodborne pathogens. Because FA treatment demonstrated the complete inactivation of EC, LM, and ST after only 1 min of treatment, the effect of dipping time on FA was not evaluated.

## 4. Discussion

Environmental temperature stress induces enhancement of the antimicrobial activity of sanitizer [1,18]. However, in the current study, the increase of temperature did not successively lead to a regular enhancement of the effectiveness of sanitization when using FA to inactive SA populations. For example, the reduction of SA did not show significant differences from 40 to 60 °C (*p* < 0.05) (Figure 1). After 1 min, FA treatment resulted in the complete inactivation of EC, LM and ST populations, whereas this activity was independent of the treatment temperature as strong sanitizing activity was also observed at low temperatures (25 and 30 °C). Issa-Zacharia *et al.* [17] reported that sodium hypochlorite (NaClO) treatment with an ACC of 120 mg/L was able to reduce the populations of SA, EC and *Salmonella* spp. by 4.91, 5.13, and 5.22 log CFU/mL, respectively, when carried out at room temperature for 1 min. However, in the current study, FA treatment (0.25%) successfully inactivated EC and ST at 25 °C when treated for 1 min, and reduced SA populations by approximately 4.86 log CFU/mL at 30 °C within 1 min. This further justifies that the hydrophobic nature of FA may make it an effective antimicrobial agent. Hydrophobicity is important because the microbial cell wall normally contains lipid material. Hydrophobic organic acids can interact with this lipid material in a way that disrupts microbial activity [13].

The effect of temperature on the efficacy of SAEW was examined to gain an understanding of the impact of temperature stress on cell behaviors when exposed to inactivation with sanitizer. EC, ST, LM, and SA were more rapidly inactivated by SAEW at 60 °C than at 40 or 50 °C, which in turn was more rapid than at 25 or 30 °C (Figure 2). It seems that the sanitizing effect of SAEW was enhanced by increasing treatment temperature at lower ACC (5 ppm). Temperature significantly affects membrane lipid composition and physical state. At ambient atmospheric temperature, lipids in biological membranes usually exist in a fluid, liquid-crystalline state [18] which provides maximal permeability and flexibility. Due to this condition, pathogens can exhibit the best resistance, which may thus be attributed to greater membrane flexibility at that temperature. At low temperatures (room temperature 23 ± 2 °C), crystallization of the phospholipids occurs, making cell membranes more rigid and consequently more sensitive to pressure. At moderately high temperatures (40–50 °C), hydrogen and hydrophobic bonds may be weakened, making bacterial membranes less resistant to conditions of temperature stress. This condition of stress may allow the HOCl to penetrate the cytoplasmic membrane and enhance its killing action.

Increasing in the concentration of free chlorine from 5 to 30 ppm resulted in a significant increase in the bacterial reduction (*p* < 0.05) of each of the examined pathogenic strains, which suggests that the free chlorine in SAEW may be the most important factor for killing pathogens [19]. In contrast, the effect of temperature did not significantly increase the rate of inactivation when compared to the effect of concentration. For example, increase of the temperature to 60 °C for 1 min of treatment did not result in bacterial inactivation of 4 and 5 log CFU/mL for SA and EC, respectively. However, when the concentration was increased to 20 ppm of free chlorine for 1 min of treatment, the bacterial inactivation reached values over 4 and 5 log CFU/mL for SA and EC. Thus, the sanitizing activity of SAEW was primarily enhanced by the free chlorine concentration, more so than by the treatment temperature. For SA*,* increase of the FA concentration to 0.5% did not reach complete inactivation even though the temperature was also increased to 40 °C. SA is becoming an increasingly important threat to the food industry and to public health worldwide because of the remarkable ability to expand its genome, and thereby, to acquire resistance mechanisms against whole classes of antibacterial agents, such as acidic stress [20]. Previous studies investigated the responses of SA when exposed to HCl and organic acid stress, and reported that SA exhibited flexible and versatile responses to different types of acid stress since it can increase the pH of the medium, mainly through the accumulation of ammonium and removal of acid groups, resulting in increased production of diacetyl (2,3-butanedione) and pyrazines [20].

Based on the dipping times of SAEW solution tested on the foodborne pathogens examined in this study, reduction occurred in the order of 5 min > 3 min > and 1 min, with more than a 7 log CFU/mL reduction achieved for EC and ST, and 5 and 6 log reductions observed for SA and LM, respectively. The principle of multiple intervention technology is to capitalize on the different weaknesses of various pathogens strains [21]. This assumption turned out to be true, because SAEW treatment became more effective when the concentration of free chlorine was increased to 20 ppm, exhibiting complete inactivation when treated for 3–5 min for all pathogenic strains examined throughout this study.

For comparison purposes, the sanitizing effects of SAEW and FA on two Gram positive bacteria (SA and LM) and two Gram negative bacteria (EC and ST) were examined under variable experimental conditions. The results revealed that SAEW treatment did not demonstrate particular differences in reduction between the Gram positive and negative bacteria. As an exception, when the pathogens were treated with SAEW at 40 °C with the free chlorine concentration of 10 ppm (Figure 5A), the treatment demonstrated significant differences in reduction between Gram positive and Gram negative bacteria. FA treatment significantly reduced both Gram positive and Gram negative bacteria (*p* < 0.05) over SAEW treatment. The lethal effects of SAEW on Gram negative bacteria were significantly different (*p* < 0.05). SA and LM were more resistant to both SAEW treatments than EC and ST, especially at lower concentrations. Issa-Zacharia *et al.* [1] also found similar results when they evaluated the sanitization potency of SAEW on EC and SA. The authors reported that the Gram positive bacterium SA was relatively more resistant than the Gram negative bacterium EC. In addition, pathogens vary in their sensitivity to sanitizers. For example, LM is generally more resistant to chlorine than *Salmonella* spp. and EC [22].

FA has high antimicrobial activity because the undissociated form of weak acids passes freely through the cell membrane. As the cytoplasmic pH is generally higher than that of the growth medium, the weak acid dissociates to release a proton, leading to acidification of the cytoplasm [23]. Studies on Gram negative foodborne pathogens, including ST and EC, have clearly demonstrated that tolerance of low pH can be induced by prior exposure of these organisms to sublethal pH conditions [24]. This assumption has also been reported for Gram positive bacteria such as LM and SA [23]. Analysis of the nature of acid adaptation in *Listeria* strains has determined that the tolerance of LM to low pH requires protein synthesis, and can induce potent cross-protection against other stresses [25]. Overall, the FA treatment was more effective than the SAEW treatment herein. The difference observed in this study may be due to the greatly different values of pH which exist between these treatments. Eklund *et al.* [26] reported that organic acids acting as inhibitors of microbial growth show a clear pH dependency: if the proton potential directly increases in the solution, the organic acid becomes more effective. However, the high pH of EW may sensitize the outer membranes of the bacterial cells to the entry of HOCl into the cells [5]. For this reason, the high pH value in SAEW may limit its potential to disrupt the outer membrane to allow the HOCl to penetrate the cytoplasmic membrane. The ability of organic acids to permeabilize the outer membrane of the tested pathogens may explain the greater effectiveness compared with SAEW.

## 5. Conclusions

This work evaluated the potential sanitizing effects of SAEW and FA under different experimental conditions. The degree of the antimicrobial effect varied to a certain extent depending on the type of pathogens or experimental conditions. In general, Gram positive bacteria were shown to be more resistant than Gram negative bacteria to all treatments throughout this experiment. FA at low concentrations (0.25%) was the most effective on EC, SA, LM, and ST inactivation, being much more effective compared to SAEW treatment. Therefore, these results suggest that FA at low concentrations (0.125%–0.25%) can be used for the effective decontamination of food products. Although the results demonstrated a difference in the bacterial reduction between treatments, both FA and SAEW treatments were effective for the reduction of all pathogens examined throughout this experiment. SAEW has limited action to disrupt the outer membrane for HOCl penetration into the cytoplasmic membrane should further be studied to find a combination with other some sanitizers which can promote HOCl penetration into the cytoplasm to improve its potential. It has been proved that SAEW could improve the physicochemical and sensory quality of different food matrix [6,9]. However, this work showed the FA antimicrobial potential using pure culture of different pathogens and we suggest more studies on its sanitization effects and also the organoleptic effect on different food products.
